# A randomized double-blind placebo-controlled trial to evaluate the value of a single bolus intravenous alfentanil in CT colonography

**DOI:** 10.1186/1471-230X-11-128

**Published:** 2011-11-23

**Authors:** Thierry N Boellaard, Marije P van der Paardt, Susanne Eberl, Markus W Hollmann, Jaap Stoker

**Affiliations:** 1 Department of Radiology, Academic Medical Center, University of Amsterdam, the Netherlands; 2 Department of Anaesthesiology, Academic Medical Center, University of Amsterdam, the Netherlands

## Abstract

**Background:**

Although CT colonography is a less invasive alternative for colonoscopy for the detection of colorectal polyps and cancer, procedural pain is common. In several studies, CT colonography pain and burden is higher than in colonoscopy. Apart from discomfort, anxiety and its related stress-induced peri- procedural side effects, this may influence the adherence for CT colonography as a possible screening tool for colorectal cancer. We hypothesize that a single bolus intravenous alfentanil will give a clinically relevant reduction in maximum pain defined as at least 1.3 point reduction on an 11-point numeric rating scale (NRS).

**Methods/Design:**

A randomized double-blind placebo-controlled trial in which patients scheduled for elective CT colonography in a single tertiary centre are eligible for inclusion. The first 90 consenting patient will be block-randomized to either the alfentanil group or the placebo group. Before bowel insufflation, the alfentanil group receives a single bolus intravenous alfentanil 7.5 μg/kg dissolved in 0.9% NaCl, while the placebo group receives an intravenous bolus injection of pure 0.9% NaCl. For both groups an equal amount of fluid per kilogram (75 μL/kg) is injected. The primary outcome is the difference in maximum pain on an 11-point NRS. Secondary outcomes include: pain and burden of different CT colonography aspects, side effects, procedural time and recovery time. For the primary outcome an independent samples t-test is performed and a P value < 0.05 is considered statistically significant.

**Discussion:**

This study will provide evidence whether a single bolus intravenous alfentanil gives a clinically relevant reduction in maximum pain during CT colonography.

**Trial registration:**

Netherlands Trial Register (NTR): NTR2902

*This trial will be conducted in accordance with the protocol and in compliance with the moral, ethical, and scientific principles governing clinical research as set out in the Declaration of Helsinki (1989) and Good Clinical Practice (GCP). The department of radiology of the Academic Medical Center of Amsterdam is responsible for the design and conduct of the trial*.

## Background

Computed tomographic (CT) colonography is an equally accurate and less invasive alternative for colonoscopy for diagnosing colorectal cancers and large- to medium-sized polyps [1-3]. The method is used in clinical practice while it has been adopted as colorectal cancer screening tool in the USA and is being considered as such in other countries [4,5]. Although CT colonography is less invasive than colonoscopy, pain is frequently observed during bowel insufflation [6-12]. Insufflation is a prerequisite for accurate visualization of the bowel wall [13]. Bowel insufflation causes stretching of the bowel and may result in colonic cramp which causes pain. In some studies pain and/or burden of CT colonography even compares unfavourably with conventional colonoscopy under conscious sedation [6-8].

Apart from the discomfort, anxiety and its related stress-induced side effects peri- procedural the, the experienced pain may well reduce the adherence of CT colonography as a potential screening tool for colorectal cancer. Especially in a screening setting - where the majority of participants does not benefit from the scan - participants will give a substantial weight to pain and burden compared with symptomatic patients who are much more likely to benefit from the scan.

During conventional colonoscopy administration of analgesics is standard. To the best of our knowledge, no analgesic is administered during CT colonography. To induce sufficient analgesia during CT colonography an opioid will be necessary. Studies using preventive oral analgesic agents for acute pain often do not result in a significant pain reduction [14-17]. In colonoscopy opioids are routinely used, often in combination with a benzodiazepine because of the synergetic effect [18,19]. CT colonography is expected to be less painful compared with colonoscopy without the administration of analgesic medication. A benzodiazepine as co-medication is therefore probably not required. Because of a procedural time of about 20 minutes a short acting opioid like fentanyl or alfentanil will be sufficient and thus prevents long recovery times [20-22]. An opioid bolus has been shown to improve pain scores during sigmoidoscopy [23]. Alfentanil has the advantage of being one of the most short-acting opioids and with the shortest recovery time [20-22]. The need for recovery facilities could have detrimental consequences on the clinical use as well as for CT colonography as a screening tool and this favours alfentanil [24].

Alfentanil is a relatively safe drug, but has possible side effects like other opioids such as: nausea, vomiting, hypotension, bradycardia and respiratory depression. However, in low- to medium-dose and without the use of a benzodiazepine, the incidence respiratory depression is extremely low [20,22,25,26]. In a study of Cho et al. a bolus injection of 10 μg/kg alfentanil did not induce any hypoxemia, desaturations or apnoeas [26].

Before considering analgesia in CT colonography, clinically relevant pain reduction, burden and acceptance, without detrimental effects on safety and cost-effectiveness should be demonstrated. For assessment of pain during a procedure, a numeric rating scale (NRS) is often used instead of a visual analogue scale (VAS), because it can be assessed verbally. We have experience with an 11-point NRS for pain assessment during our CT colonography procedure and therefore these data can serve as pilot data. A reduction of 1.3 points on an 11-point NRS is considered the minimum clinically relevant pain reduction [27-29]. To evaluate the effect of an intervention with medication such as alfentanil in CT colonography, a randomized (placebo) controlled trials is the optimal study design. As alfentanil is dissolved in a 0.9% saline solution, an ideal placebo would be 0.9% saline, above all because the colour and viscosity is similar.

We therefore perform a randomized double-blind placebo-controlled trial to evaluate the effect of a single bolus intravenous alfentanil of 7.5 μg/kg on the pain during CT colonography. We hypothesize that a single bolus intravenous alfentanil will give a clinically relevant reduction in maximum pain defined as at least 1.3 point reduction on an 11-point NRS. To the best of our knowledge no previous study evaluated the use of analgesia in CT colonography.

## Methods/Design

### Objectives

#### Primary objective

To evaluate whether a single intravenous alfentanil bolus (7.5 μg/kg) has a clinically relevant analgesic effect in patients undergoing elective CT colonography compared with placebo. We have defined a clinically relevant effect as a pain reduction of 1.3 point on an 11-point NRS [27,29].

#### Secondary objectives

To assess the difference in:

• Pain score in all insufflation positions (right and left decubitus, supine and prone) and the average pain score

• Pain and burden of all CT colonography aspects (bowel preparation with oral iodinated contrast and diet, cannula insertion, rectal catheter insertion, insufflation, positional change on table and period after the procedure), and total pain and burden of CT colonography

• Side effects of alfentanil during CT colonography including:

○ respiratory effects (apnoea, respiratory frequency, and blood oxygenation)

○ hemodynamics (heart rate and blood pressure)

• Procedure and recovery time

• The most painful and most burdensome aspect of CT colonography

### Trial design

This study will be a single-centre randomized double-blind placebo-controlled trial.

### Population

Consecutive 90 patients between 18 and 85 years from the Academic Medical Center (AMC) of the University of Amsterdam, who give informed consent, will be included. The population in the AMC scheduled for CT colonography is a mixed group of sexes, ethnic backgrounds, and mostly elderly patients.

### Exclusion criteria

• Hypotension (systolic blood pressure < 90 mmHg)

• Bradycardia (heart rate < 50 beats per minute)

• Severe chronic obstructive pulmonary disease

• Known allergy for alfentanil

• Pregnancy (if the patient indicates any chance of being pregnant, this will be tested)

• Known severe liver disease defined as a Child-Pugh score of > 4

• Use of MAO-inhibitors or within two weeks before the CT colonography procedure

• Use of barbiturates, opiates or daily benzodiazepine use

• Known increased intracranial pressure

The number of excluded patients and the reasons for their exclusion will be recorded and also reported in the following article.

### Sample size

The sample size calculation was aimed at the detection of a difference in pain score between the alfentanil and placebo group. We calculated average pain score and standard deviation of a population screening trial performed in the AMC where the same insufflation procedure and 11-point NRS were used. The standard deviation was 2.6 in the pilot data. Based on our clinical experience with alfentanil we assume alfentanil will cause a reduction of 1.5 on an 11-point NRS. We tested this with a one-sided test, using 0.05 significance and 80% power. An independent samples t-test to compare means in two groups in nQuery Advisor 7.0, results in groups of 38 subjects. With an anticipated withdrawal rate of approximately 5%, we aim to include 45 patients per group.

### Informed consent

**Figure 1:**
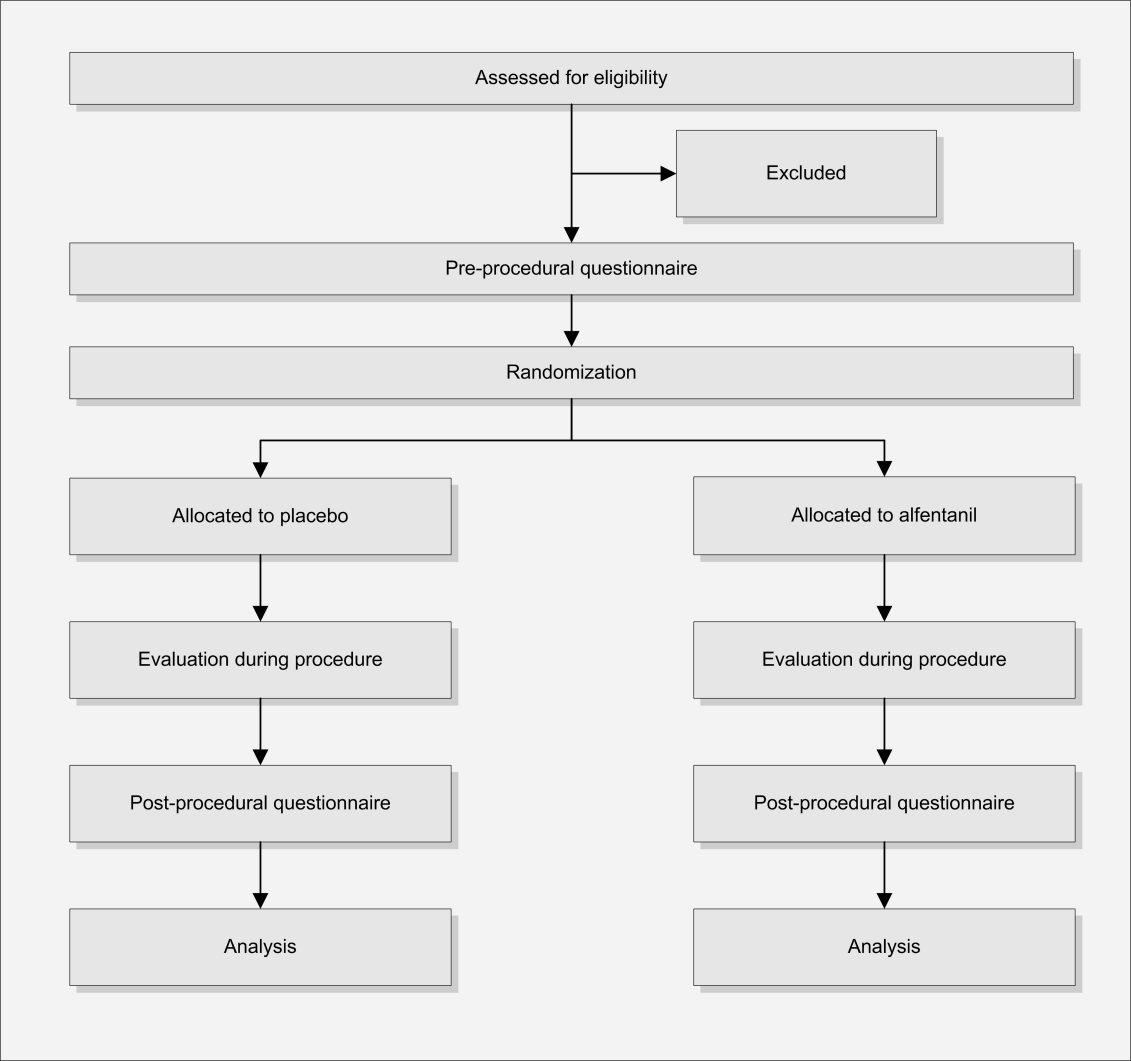
**Flow chart according to the CONSORT statement 2010**.

Patients scheduled for an elective CT colonography procedure will be asked by telephone to participate [Figure 1]. The exclusion criteria will be checked during this conversation and if they are eligible for inclusion and interested in participating in our study, the patient information will be sent. Patients are included after written informed consent. Patients may decide to participate in our study until two days before CT colonography.

### Intervention

Subjects randomized to group 1 will receive alfentanil (Rapifen, Janssen-Cilag, Tilburg, the Netherlands) 7.5 μg/kg intravenously through a 20 Gauge intravenous cannula. Subjects randomized to group 2 will receive a placebo, in this study a 0.9% saline solution 75 μL/kg through a 20 Gauge intravenous cannula. For both groups an equal amount of fluid (75 μL/kg) will be injected. This placebo is chosen because alfentanil is dissolved in 0.9% saline solution. After administration of the spasmolytic agent the line is flushed with 5 mL 0.9% and again after administration of the study medication. Both the physician performing the CT colonography scans as well as the patient are blinded to the allocated group. Oxygen saturation, heart rate, and blood pressure will be measured during the CT colonography procedure, using a pulse oximeter and automated blood pressure monitor. A pain evaluation will be performed during insufflation in left decubitus, right decubitus, supine and prone position (see section pain evaluation).

Two questionnaires with 22 and 17 questions, mostly multiple-choice, will be given during this study: the first before randomization and the second after completion of CT colonography.

### CT colonography

Both preparation and insufflation are performed as used in current clinical practice in our institution. The preparation for CT colonography consists of two bottles of 50 mL iodinated contrast, meglumine ioxithalamate (Telebrix, Guerbet, Aulnay sous Bois, France), the day before and one bottle in the morning of CT colonography and a low-fibre diet for these days [30]. Colonic distension will be obtained by the automated administration of carbon dioxide (PROTOCO2L, Bracco, EZEM, Lake Success, USA) through a flexible 20 French rectal tube after intravenous administration of 1 mL (20 mg) butylscopalamine bromide (Buscopan, Boehringer-Ingelheim, Ingelheim, Germany) or, if contraindicated 1 mL (1 mg), Glucagon (GlucaGen, Novo Nordisk A'S, Bagsvaerd, Denmark). Insufflation takes place in three positions: right decubitus, left decubitus, and supine position. The aim is to insufflate three litres of carbon dioxide with 1.3, 0.9, and 0.8 litres per position, respectively. After five minutes the insufflation is stopped, whether the target of three litres is reached or not. The scan is performed using a 64-slice CT scanner (Brilliance, Philips Medical Systems, Best, the Netherlands) using dose modulation. A scan is performed in both supine and prone position. In case of clinical suspicion of colorectal cancer, 100 mL intravenous iodinated contrast agent, iopromide (Ultravist 300, Bayer B.V., Mijdrecht, the Netherlands) will be administered in supine position. Otherwise both series are unenhanced.

Parameter unenhanced CT: 120 kV

40 reference mAs

64 × 0.625 collimation

0.9 mm slice thickness

Parameters contrast-enhanced CT: 120 kV

200 or 250 reference mAs

64 × 0.625 collimation

0.9 mm slice thickness

### Analgesia

One of the two independent research physicians, who are in control of the randomization list, will prepare the alfentanil or the placebo. Subsequently, a blinded qualified physician, who performs the CT colonography, will infuse the medication/placebo via the intravenous cannula over a period of approximately two minutes. The medication/placebo is given 1.5 minutes after butyl scopolamine or glucagon is given and the average blood pressure and heart rate has been recorded. Butyl scopolamine influences the heart rate and blood pressure. After 1.5 minutes the effect of butyl scopolamine on the heart rate and blood pressure is present and a reliable baseline value can be registered.

### Monitoring

During the whole procedure, the patients will be monitored using a pulse oximeter. An automatic blood pressure device will monitor the blood pressure before administration of the spasmolytic agent and 1.5 minutes after the spasmolytic agent is given intravenously. After the first blood pressure measurement the blood pressure is automatically measured and recorded every five minutes. The heart rate and oxygen saturation is continuously recorded by pulse oximetry.

### Pain evaluation

During the procedure pain is assessed immediately after insufflation in left decubitus, right decubitus, supine and after scanning in prone position using an 11-point NRS. This score starts at 0, meaning no pain at all and ends at 10 means the worst pain imaginable. The score therefore contains 11 points. All four pain scores will be reported.

### Time

The begin time and end time of the CT colonography procedure will be noted. The begin time is defined as the moment the patient enters the CT scanner room after changing clothes. The end time is defined as the moment the patient leaves the CT scanner room after the CT scan has been performed. Additionally, the time from the start of the procedure until the end time of recovery is noted. The end of recovery is defined as the time that the first Aldrete score of 9 or higher is measured.

### Questionnaires

All patients scheduled for clinical CT colonography will receive patient information and an informed consent form. Along with these forms, a pre-procedural questionnaire is sent to assess the baseline characteristics and expectations:

• Baseline characteristics include: age, gender, marital status, ethnicity, and education

• Expectations include burden and pain of: the bowel preparation, intravenous cannula insertion, bowel insufflation, and the total procedure on a standard formatted 5-point scale (no pain, mild pain, moderate pain, severe pain, and very severe pain)

A post-procedural questionnaire is given 30 minutes after the procedure. This time is standardized because the time between the procedure and the questionnaire may influence the answers. This questionnaire is designed to assess the experienced burden and acceptability:

• Experience includes burden and pain of: the bowel preparation, intravenous cannula insertion, bowel insufflation, and the total procedure on standard formatted 5-point scale (identical to the pre-procedural form)

• Which aspect of the procedure was most painful or burdensome (bowel preparation, intravenous cannula insertion, rectal catheter insertion, bowel insufflation, turning on the examination table or symptoms after examination)

• Acceptance includes: if they would accept this as a method for population screening

Both the pre-procedural questionnaire and post-procedural questionnaire are based on questionnaires used in a previous CT colonography trial [4].

### Adverse events

Serious adverse event as defined by the ICH Guidelines for Good Clinical Practice E6 will be recorded and reported and mild adverse events, such as nausea will be recorded.

### Safety

An oxygen mask, an AMBU set, and naloxone will be available on site. The research physician, who prepares the study medication, will leave a signed sealed envelope (with patient name and patient number) with the given medication, which can be opened in case a serious adverse event occurs. The most serious adverse event caused by alfentanil within this setting is respiratory depression. The person administering alfentanil is trained in advanced life support; ensuring that this individual is competent in diagnosing respiratory depression and performing the appropriate treatment. In case of respiratory depression the patient will be ventilated, using a bagging bag with mask. Therefore oxygen, mask, and a bagging bag will be nearby.

To antagonize the induced respiratory depression 0.2-0.4 mg naloxone (B. Braun, Melsungen, Germany) will be given if necessary and can be repeated every 2-3 minutes. As extra precaution, a rescue plan has been created describing all steps that need to be performed in case of respiratory depression, hypotension, and bradycardia. The anaesthesiologists on call for emergency situations are informed about all CT colonography exams that will be performed.

### Recovery criteria

**Figure 2:**
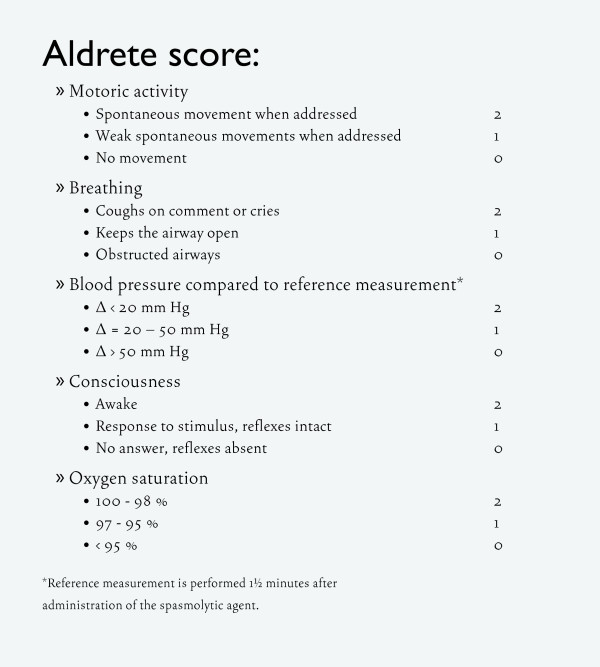
**Aldrete recovery criteria**.

Each patient has to be observed by a blinded qualified staff member for one hour after the CT colonography procedure. For the monitoring of the recovery, the Aldrete score [31], a very commonly used score for recovery monitoring, is used at arrival at the recovery room and at 30 min and 60 min after alfentanil administration [Figure 2]. A score of 9 or higher obtained at 60 minutes is considered as ready for discharge and the participant may go home. If the score is lower than 9, another Aldrete score will be performed at 90 and 120 minutes. All scores will be recorded.

### Statistical analysis

SPSS version 16.0 is used for all statistical calculations. A P value of 0.05 is defined as significant. Baseline characteristics are summarized with descriptive statistics. For categorical data the numbers or proportions are given, for normal distributed data the mean and standard deviation and for not normal distributed data the median with percentiles.

Differences between the alfentanil group and the placebo group will be tested for significance. For the calculation of differences in pain scores per position, average, and the maximum pain score during the procedure will be calculated using an independent samples t-test.

Differences in burden of different aspects of the procedure as well as the total procedural burden will be calculated using a chi-square test. Differences in the most burdensome aspect will also be calculated using the chi-square test. Differences in procedural time and recovery time will be calculated using an independent samples t-test.

We will test the associations between the baseline characteristics (independent variables mentioned below) and pain scores. As we consider the pain scores as continuous data, for this approach we will perform linear regression analyses.

Univariate analyses will be performed with pain scores as dependent variable and the following variables as independent variables: age, sex, BMI, education, ethnicity, expected burden, scan indication (including abdominal pain), known diseases and diagnosis, and whether it is the first CT colonography. Subsequently, the variables with a P value < 0.1 will be included in the multivariate analysis. In case more than four variables turn out to result in a P < 0.1, the four most influential variables will be used in the multivariate analysis. A stepwise backward selection strategy will be used, with a P value < 0.05 considered statistically significant.

### Ethical approval

Ethical approval was obtained from the Medical Ethics Committee of the Academic Medical Center, Amsterdam, the Netherland (NL35916.018.11). A marginal review was performed by the National Authority, the Central Committee on Research Involving Human Subjects (CCMO), and there were no objections to perform this study (NL35916.018.11 BI).

## Discussion

CT colonography is a structural examination of the colon and rectum. It is used in high risk patients as an alternative for colonoscopy or in case colonoscopy is incomplete or contraindicated. Although CT colonography is less invasive, results on pain and burden during these examinations in literature are variable. Several studies show higher procedural pain and burden for CT colonography compared with colonoscopy with conscious sedation due to air or carbon dioxide insufflation [6-8]. With an appropriate analgesic agent, CT colonography may not only be less invasive, but also less painful and less burdensome, which would be advantageous for patients.

Additionally, the screening test for colorectal cancer is still under debate. Several screening tools are available such as faecal occult blood test, sigmoidoscopy, colonoscopy, and CT colonography. The effectiveness of a screening tool is influenced by both participation and yield. Colonoscopy and CT colonography are the most sensitive techniques for the detection of colorectal neoplasia, i.e. colorectal cancer and its precursor, colorectal advanced adenomas [32]. The advantages of colonoscopy are the highest sensitivity and opportunity for direct polyp removal. Advantages of CT colonography are that this technique is less invasive and has a very low complication rate [33]. CT colonography adherence and participation most likely will be influenced by a clinically relevant reduction in pain and burden experienced during the examination and therefore may have impact on the principal outcome measure of colorectal cancer screening, i.e. the number of advanced neoplasia per 100 invitees.

This RCT will provide evidence whether a single bolus intravenous alfentanil gives a clinically relevant reduction in maximum pain during CT colonography. Furthermore, this study will provide information about the effect of alfentanil on pain and burden of different CT colonography aspects, side effects, adverse events, procedural time and recovery time. We expect a clinically relevant reduction of procedural pain and burden without adverse events and recovery time. This could make CT colonography a more patient-friendly examination and is likely to increase participation for its use as a screening tool.

## Abbreviations

CT: computed tomography; NRS: numeric rating scale; BMI: body mass index; NaCl: sodium chloride; kV: kilo volts; mAs: milliampere second; ICH: The International Conference on Harmonisation of Technical Requirements for Registration of Pharmaceuticals for Human Use; RCT: randomized controlled trial; AMC: Academic Medical Center.

## Competing interests

The authors declare that they have no competing interests.

## Authors' contributions

TNB is responsible for drafting the manuscript. TNB, MPP, SE, MWH, and JS are responsible for the study design. MPP, SE, MWH and JS are responsible for revising the manuscript. All authors have read and approved the manuscript.

## Pre-publication history

The pre-publication history for this paper can be accessed here:

http://www.biomedcentral.com/1471-230X/11/128/prepub
